# Increased Antioxidant Performance of Lignin by Biodegradation Obtained from an Extract of the Mushroom *Pleurotus eryngii*

**DOI:** 10.3390/molecules29235575

**Published:** 2024-11-26

**Authors:** Tania Petraglia, Tiziana Latronico, Antonietta Pepe, Aniello Crescenzi, Grazia Maria Liuzzi, Rocco Rossano

**Affiliations:** 1 Department of Basic and Applied Sciences, University of Basilicata, 85100 Potenza, Italy; tania.petraglia@unibas.it (T.P.); antonietta.pepe@unibas.it (A.P.); 2 Department of Biosciences, Biotechnologies and Environment, University of Bari “Aldo Moro”, 70126 Bari, Italy; tiziana.latronico@uniba.it; 3 Department of Agricultural, Forestry, Food and Environmental, University of Basilicata, 85100 Potenza, Italy; aniello.crescenzi@unibas.it

**Keywords:** lignin, antioxidants, laccases, biodegradation, ROS, DI-TNC1 cell line

## Abstract

The aim of this study was to evaluate the antioxidant properties of the products derived from the biodegradation of lignin by the ligninolytic enzymes present in an aqueous extract of the mushroom *P. eryngii*. A mixture obtained after the incubation of lignin for 18 h with *P. eryngii* extract was tested in vitro for its total polyphenol content, reducing power, and 1,1-diphenyl-2-picrylhydrazyl (DPPH) and hydroxyl (OH) radical-scavenging activities. The results showed that the enzymatic treatment of lignin enhanced its antioxidant performance. The biocompatibility of the products of lignin biodegradation and their ability to scavenge reactive oxygen species (ROS) were also tested on the astrocytic cell line DI-TNC1. The results obtained indicated that a lignin mixture incubated for 18 h does not affect cell viability or inhibit the H_2_O_2_-induced ROS production. These results suggest that the enzymatic degradation of lignin represents an efficient and ecofriendly approach to obtain lignin derivatives potentially useful for antioxidant applications.

## 1. Introduction

Lignin is one of the main constituents of lignocellulosic biomass; it is the second most abundant natural polymer after cellulose. Lignin is an amorphous hydrophobic polymer with a very complex cross-linked structure that varies greatly depending on the plant species of origin. Lignin results from the polymerization of three monolignols, namely coniferyl alcohol (G), sinapyl alcohol (S), and p-coumaryl alcohol (H) [1,2,3]. Due to its aromatic nature, the depolymerization of lignin produces various phenolic compounds with numerous biological properties [4,5,6,7]. Among them, one of the most important biological activities is its antioxidant properties, attributed to the ability of its phenolic structures to reduce free radicals through hydrogen or electron transfer [8,9]. However, its aromatic nature and its complex cross-linked structure, as well as the consequent poor solubility of lignin, make it chemically difficult to degrade [10]. In recent years, the depolymerization of lignin to produce high value-added chemical compounds has represented one of the most important challenges [11,12,13]. A highly successful strategy to promote the degradation of lignin into biologically active products is represented by enzymatic treatment. Compared to thermal or chemical degradation, the enzymatic depolymerization of lignin, and therefore its valorization through a biological approach, could guarantee both economic and environmental benefits represented by a reduction in the use of toxic chemicals and the use of renewable and inexpensive biological catalysts. In addition, the biological valorization of lignin would represent an interesting opportunity for the pharmaceutical and biomedical fields [14,15,16,17,18].

It is known that many fungi and some bacterial species are very efficient in the enzymatic degradation of lignin [19,20]. Bacteria are less efficient in lignin depolymerization than fungi. Among the latter, white rot fungi (e.g., *Ganoderma* spp., *Lentinula edodes* or *Pleurotus* spp.) are the most efficient lignin degraders. This ability is due to the combined action of two groups of enzymes, such as phenol oxidase (laccase, EC 1.10.3.2) and peroxidases (lignin peroxidase, EC 1.11.1.14; manganese peroxidase, EC 1.11.1.13; versatile peroxidase and dye-decolorizing peroxidase, EC 1.11.1.19) [21,22]. Due to their high redox potential, laccases from ligninolytic fungi are considered the preferred enzymes for the enzymatic depolymerization of lignin into phenolic groups and chemicals of biological origin [23,24]. In most of the studies conducted on the enzymatic depolymerization of lignin, purified enzymes or a mixture of them is generally used [25,26,27]. An interesting aspect of this work consists of having used an aqueous extract of the mushroom *P. eryngii*, which offers several advantages when compared to the commercial purified ligninolytic enzymes In fact, the aqueous extract used in this study is characterized by the presence of various ligninolytic enzymes, the simplicity of preparation, and the low costs of production compared to those incurred for the purification of commercial enzymes.

In the experiments of this study, the extract from *P. eryngii* was used to evaluate the antioxidant properties of products derived from the enzymatic biodegradation of lignin extracted from the shell of pecan nut. The results showed that the enzymatic treatment of lignin improved both its antioxidant performance in vitro and its ability to scavenge reactive oxygen species (ROS) in a cell-culture model represented by DI-TNC1 cells.

## 2. Results and Discussion

### 2.1. Lignin Characterization

**Figure 1:**
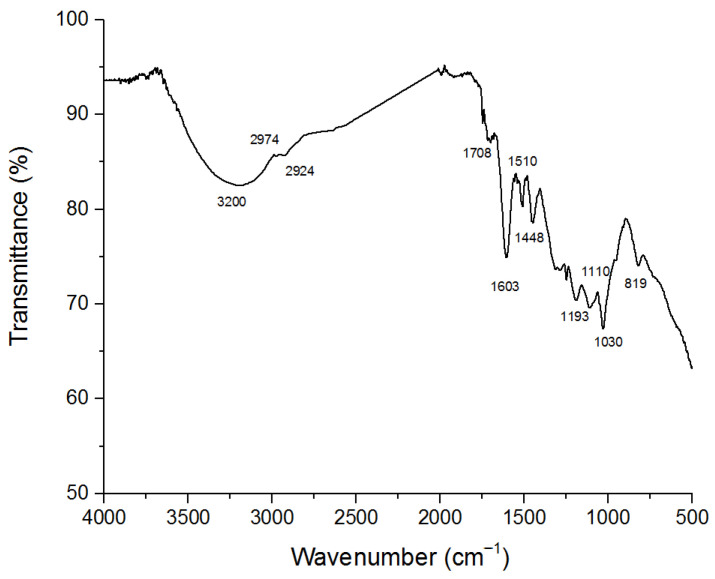
The ATR-FTIR spectrum of lignin extracted from pecan shells. The spectral analysis is described in the text.

To characterize the structure of lignin from pecan shells, Attenuated Total Reflectance–Fourier Transform Infrared (ATR-FTIR) Spectroscopy was performed, with the results compared to the values reported in the literature [28,29]. The ATR-FTIR spectrum was recorded in the 500–4000 cm^−1^ region and revealed the presence of functional groups in different regions. It shows typical absorptions of aromatic molecules at 1603 and 1510 cm^−1^ assigned to the C=C stretching vibration, while the absorption at 1448 cm^−1^ is due to the bending vibration of methoxy groups of guaiacyl (G) and syringyl (S) units. The band at 1708 cm^−1^ was assigned to the stretching of carbonyl groups (C=O) in unconjugated ketones, conjugated aldehydes, and aromatic carboxylic acids. The band at 1110 cm^−1^ is assigned to the aromatic C-H in plane deformation of S units, while the band at 1030 cm^−1^ could be assigned to guaiacyl units. Finally, the band at 819 cm^−1^ was ascribed to the out-of-plane deformation of the aromatic rings of both S and G units (Figure 1).

### 2.2. Total Phenolics and Educing Apacity

**Figure 2:**
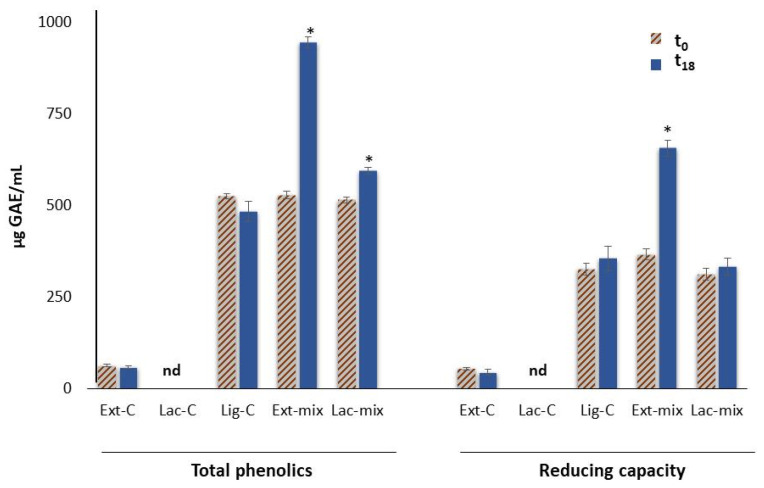
The total phenolics and reducing capacity detected in vitro in lignin solutions after enzymatic treatment with laccase (Lac-mix) or *P. eryngii* extract (Ext-mix), and with the corresponding controls: Lac-C, Ext-C, and Lig-C. The results are expressed as μg of gallic acid equivalents (GAEs)/mL of sample. Asterisks indicate a statistically significant difference in comparison to the baseline value (t_0_) (one-way ANOVA followed by Tukey’s post hoc test; *p* < 0.05).

Figure 2 shows the phenolic content and the reducing capacity (of lignin solutions after enzymatic treatment with laccase (Lac-mix) or with the mushroom *P. eryngii* extract (Ext-mix), and the corresponding controls: Lac-C, Ext-C, and Lig-C, respectively. The total phenolic content and reducing capacity of samples were expressed as µg of gallic acid equivalents (GAEs)/mL.

As shown in Figure 2, Ext-C had a very low phenolic content compared to the other samples analyzed. In fact, to evaluate the release of phenolic compounds derived from the depolymerization of lignin by ligninolytic enzymes, the endogenous polyphenols contained in the *P. eryngii* extract (1.4 mg of gallic acid equivalents (GAEs)/mL) were removed by filtration on filters with a cut-off of 10 kDa. The results indicated that after 18 h of enzymatic treatment, the phenolic contents of lignin solutions were increased by 79.0% for Ext-mix and by 15.5% for Lac-mix, respectively. Whereas, in the case of the reducing power, a significant increase (79.4%) was observed only for the lignin treated with the mushroom extract (Ext-mix). By contrast, in Ext-C, Lac-C, and Lig-C, no change was detected after 18 h of incubation compared to t_0_. Similarly, no change was detected after 18 h of incubation of lignin with the extract subjected to thermal treatment (^tt^Ext-mix). The values of phenolic content and the reducing capacity of all the analyzed samples are listed in Table 1.

These results indicated that the enzymatic treatment contributed to the increase in phenolic content in lignin due to its depolymerization, in agreement with what was reported by other authors [4]. In this regard, however, it must be pointed out that most of the studies concerning the enzymatic depolymerization of lignin have been carried out using purified enzymes and, in particular, laccases [4]. The fact that the treatment of lignin with the mushroom extract determined a greater increase in phenolic compounds compared to the treatment with laccase could be the result of a synergic action of laccases with other fungal ligninolytic enzymes, such as the peroxidases present in the extract from *P. eryngii* [21,22]. In our experiments, this hypothesis was confirmed by measuring the activity of laccases and peroxidases, which evidenced the presence of the two enzymes in the extract (Table 2).

### 2.3. Antioxidant Activity

**Figure 3:**
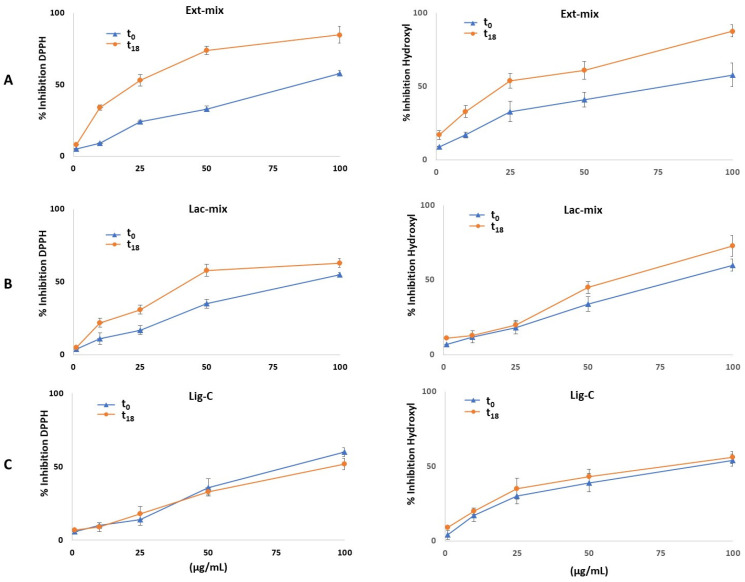
Antioxidant activity in vitro. The graphs represent the scavenging activities of lignin against the DPPH (left panel) and hydroxyl (right panel) radicals. (**A**): The lignin solution after enzymatic treatment with *P. eryngii* extract (Ext-mix); (**B**): the lignin solution after enzymatic treatment with laccase (Lac-mix); and (**C**): lignin solution control (Lig-C).

In this study, the antioxidant activity of the lignin solutions was evaluated by DPPH and hydroxyl radical-scavenging assay. The results are expressed as IC_50_ values that represent the concentration of antioxidant necessary to scavenge 50% of free radicals. As shown in Figure 3 and as listed in Table 1, the enzymatic treatment of lignin for 18 h (graphs A and B, corresponding to Ext-mix and Lac-mix, respectively), determined a dose-dependent increase in its antioxidant activity towards both radicals, compared to untreated lignin (graph C, corresponding to Lig-C). In particular, lignin treated with *P. eryngii* extract (Ext-mix) exhibited the strongest antioxidant activity (IC_50_ = 27.4 ± 3.7 μg/mL) toward the DPPH radical compared to that treated with laccase (Lac-mix, 62.5 ± 2.8 μg/mL) and the untreated one (Lig-C, 92.8 ± 2.1 μg/mL). Similar to the hydroxyl radical, the enzymatic treatment of lignin leads to an increase in its antioxidant capacity, greater for Ext-mix (IC_50_ = 35.9 ± 4.4 μg/mL) than for Lac-mix (IC_50_ = 64.3 ± 2.9 μg/mL). However, as listed in Table 1, it should be noted that both the DPPH and the hydroxyl radical-scavenging ability of Ext-mix were lower than those of the antioxidant gallic acid (IC_50_ = 5.3 ± 0.3 and 3.4 ± 0.2 μg/mL, respectively) used as the positive control.

The increase in the antioxidant capacity of enzymatically treated lignin was consistent with the increase in the total phenol content (Figure 2) responsible for the generation of new aromatic -OH groups, in agreement with the results reported by other authors [8]. To this end, Dizhbite et al. [30], investigating the structure–activity relationship of lignins isolated from different wood species and lignin-related monomeric compounds by DPPH assay, demonstrated that the antioxidant activity of lignin was attributed to the non-etherified phenolic hydroxyl groups, the ortho-methoxy groups, and the aliphatic hydroxyl groups in the side chain.

### 2.4. Effect of Lignin on DI-TNC1 Viability

**Figure 4:**
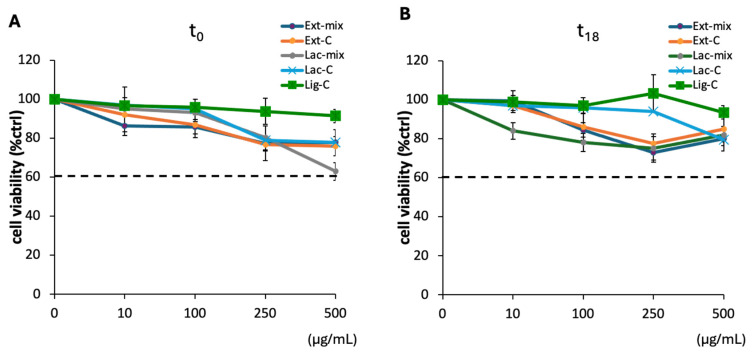
The effect of the different lignin solutions on cell viability. Confluent DI-TNC1 cells were treated with the Ext-mix and Lac-mix, as well as with the controls Ext-C, Lac-C, and Lig-C, both at t_0_ (**A**) and after incubation for 18 h (**B**). After 20 h of incubation at 37 °C and 5% CO_2_, DI-TNC1 were subjected to the MTT assay. The results are expressed as the percentage of surviving cells over untreated cells. Data are presented as mean ± SD of three different experiments with independent cell populations. The horizontal dashed line, set at 60%, indicates the threshold of cell viability. Concentrations of the samples that yielded cell viability values < 60% were considered as toxic doses.

The antioxidant properties of lignin, increased following treatment with the ligninolytic enzymes contained in *P. eryngii* extract, make it potentially usable in various fields, including biomedical research, cosmetics, polymer materials, and food. However, the practical application of lignin and its derivatives requires an evaluation of its biocompatibility in order to ensure its safe use. To assess the biocompatibility of Ext-mix, we evaluated cell viability on the DI-TNC1, a cell line established from primary cultures of type 1 astrocytes from murine brain tissue [31]. This cell line has already been used in a previous work to evaluate the cytotoxicity of a *P. eryngii* extract enriched in polysaccharides [32]. As reported in Figure 4, no cytotoxicity was evidenced for Ext-mix at all the concentrations tested, both at t_0_ (Figure 4A) and after incubation for 18 h (Figure 4B). Similarly, both the controls and the lignin treated with laccase (Lac-mix), incubated under the same experimental conditions, showed no cytotoxicity.

### 2.5. Effect of Treated Lignin on ROS Production

**Figure 5:**
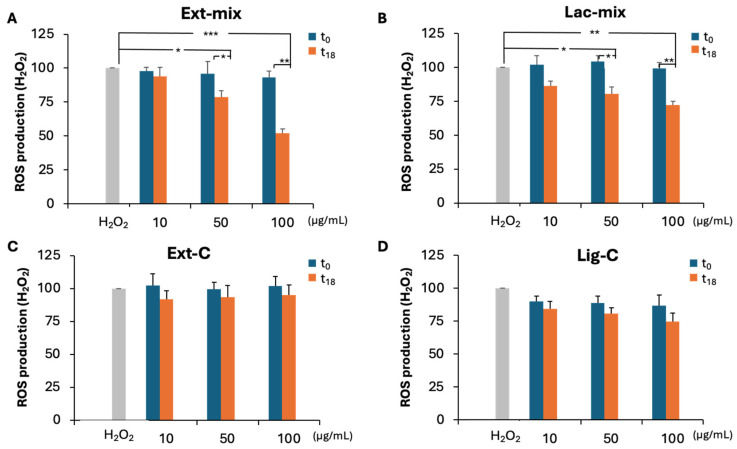
The production of reactive oxygen species (ROS) in DI-TNC1 cells treated with lignin solutions. The presence of ROS was assayed, measuring the changes in the fluorescent signal of 2′,7′-dichlorofluorescein (DCFA) as reported in the Materials and Methods section. Confluent DI-TNC1 cells, seeded in 96-well plates, were pre-treated for 2 h with the Ext-mix (**A**) and Lac-mix (**B**), as well as with the controls Ext-C (**C**), Lac-C, and Lig-C (**D**) at the indicated concentrations, then loaded with 10 μM of the fluorescent probe 2′,7′-dichlorofluorescein diacetate (DCFH-DA). After 30 min of incubation, H_2_O_2_ at a final concentration of 500 μM was added to the wells. DI-TNC1, treated with DCFA alone (CTRL) or with 500 μM H_2_O_2_, represented the negative and positive control, respectively. The fluorescence was measured by a fluorometer at 525 nm under excitation at 485 nm. The ROS production was expressed as a percentage (%) of the photoluminescence (PL) intensity in comparison to the positive control. Values are the mean ± SD of n = 3 experiments performed on different cell populations. A statistically significant decrease in comparison with H_2_O_2_ is indicated by asterisks (one-way ANOVA followed by Dunnet’s post hoc test; * *p* < 0.05; ** *p* < 0.01; *** *p* < 0.001).

We also evaluated the ability of Ext-mix and Lac-mix to counteract H_2_O_2_-induced ROS production in the DI-TNC1 cell line. As reported in Figure 5, a dose-dependent decrease in ROS production was observed in t_18_ samples of Ext-mix (Figure 5A) and Lac-mix (Figure 5B) but not in the corresponding controls (Figure 5C,D). By contrast, no protective effect was observed at t_0_ for all the preparations tested. A statistically significant decrease in ROS production was detected at the concentrations 50 and 100 μg/mL in Ext-mix, as well as in Lac-mix at t_18_, in comparison to H_2_O_2_ and to the corresponding samples at t_0_. In particular, as shown in Figure 5A,B, at the highest concentration tested, Ext-mix was able to reduce the production of ROS by 55% compared to the 30% for Lac-mix. This last result confirms the synergic action of laccases and peroxidases present in the *P. eryngii* extract in enhancing the performance of the biodegradated lignin. The ability to counteract ROS production exhibited by Ext-mix but not by Ext-C, suggests that this effect is due to the presence of antioxidant compounds released by lignin following the degradation by ligninolytic enzymes contained in the *P. eryngii* extract, and not by endogenous antioxidant compounds of the mushroom such as polysaccharides or other low molecular weight molecules as vitamins and polyphenols [32,33]. In fact, although in a previous work the ability of polysaccharides of *P. eryngii* extract to counteract the production of ROS in the same cell line has been demonstrated [32], it should be emphasized that the experimental conditions used to prepare the extract used in this study did not allow for the extraction of the polysaccharide fraction. Furthermore, regarding the possible contribution of low molecular weight compounds, this can be excluded, since these compounds were removed from the extract following a passage through centrifugal filters with a cut-off of 10 kDa.

## 3. Materials and Methods

### 3.1. Preparation of Enzymatic Extract

*Pleurotus eryngii* (strain BIO175) mushroom, axenically cultivated in a laboratory, was provided by Bioagritest research center (Interregional Center for Plant Diagnosis, Pignola, Italy). Fruiting bodies were freeze-dried and powdered in a mortar with liquid nitrogen, then stored at −80 °C. Crude extract was obtained by cold homogenizing, for 4 h, 1 g of powder with 15 mL of 50 mM sodium acetate buffer pH 5.5. After centrifugation (12,000× *g*, 10 min at 4 °C), the supernatant was filtered on Whatman 3 paper disks; then, 6 mL of filtrate was concentrated 10-fold in a Vivaspin 6 centrifugal filter (MWCO 10,000; GE Healthcare, Milan, Italy) at 5000× *g* and 4 °C, and washed with extraction buffer.

### 3.2. Enzymatic Laccase Assay

Laccase activity was measured by the method developed by Setti et al. [34], based on the oxidative coupling reaction between the 3-methyl 2-benzothiazolinone hydrazone (MBTH) and guaiacol (Sigma Aldrich, St Louis, MO, USA), with some modifications. Briefly, samples were prepared by adding 40 μL of extract and 20 μL of guaiacol to 1 mL of 50 mM acetate buffer at pH 5.5, preheated to 30 °C, then incubated at 30 °C for 10 min. Subsequently, 200 μL of 0.05% MBTH was added to the reaction mixture. After 7 min, the reaction was stopped by adding 200 μL of 1N H_2_SO_4_ and 400 μL of acetone, and the absorbance was measured at 502 nm (Ultrospec 2000 spectrophotomer, Pharmacia Biotech, Uppsala, Sweden). The laccase activity was expressed in terms of International Unit (U), where 1 U (μmol/min) is defined as the amount of the enzyme that catalyzes the conversion of one micromole of substrate per minute.

### 3.3. Enzymatic Peroxidase Assay

The activity of peroxidase was assessed by monitoring the oxidation of syringaldazine (Sigma Aldrich, St Louis, MO, USA) at 530 nm (*ε* = 6.5 × 10^4^·M^−1^·cm^−1^) [35]. The assay mixture (1 mL) contained 50 mM potassium phosphate buffer (pH 7.0), 0.1 mL of enzyme extract, 50 µM syringaldazine, and 4 mM H_2_O_2_. Peroxidase activity was monitored by measuring the increase in absorbance at 530 nm for 5 min. Enzyme activity was expressed in International Unit (U/mL); one Unit was defined as 1 μmol of syringaldazine oxidized per minute at 30 °C. The blank contained all solutions except the enzyme.

### 3.4. Lignin

Lignin from pecan shells was recovered by green methods using a deep eutectic solvent (DES)-based treatment, as previously reported [36].

#### 3.4.1. Chemical Characterization by Attenuated Total Reflectance–Fourier Transform Infrared (ATR-FTIR) Spectroscopy

ATR-FTIR spectra were recorded on a model J-460 instrument (Jasco Europe Srl, Cremella, Italy) equipped with an ATR accessory, a Smart Orbit with a type II A diamond crystal and a refractive index of 2.4, and a KBr beam splitter and a MCT/B detector. Spectra were recorded in the region from 4000 to 500 cm^−1^ with a spectral resolution of 2 cm^−1^ and 256 scans. Background spectrum was recorded and subtracted from the sample spectra. The spectrum was smoothed and fitted to an automatic baseline correction using Jasco Spectra Manager software ver. 154A.

#### 3.4.2. Lignin Solution

A total of 25 mg of lignin was dissolved in 1 mL of sodium hydroxide aqueous solution (0.1 M) and, after stirring for 10 min, the solution was diluted with 9 mL of 50 mM sodium acetate buffer pH 5.5/0.05% Tween 80, then centrifuged (10,000× *g*, 15 min at 4 °C) and kept in the dark at room temperature.

### 3.5. Enzymatic Treatment of Lignin with Extract or Laccase

To 0.1 mL of enzymatic extract or commercial laccase (from *Agaricus bisporus*, Sigma Aldrich, St Louis, MO, USA), both containing 21.3 U of laccase, 0.9 mL of 0.25% (*w*/*v*) lignin solution was added, which was then vortexed and incubated at 30 °C for 18 h in the dark. After incubation, the reaction mixtures (Ext-mix and Lac-mix, respectively) were centrifuged, and the supernatants were immediately used for the analysis or freeze-dried. In another set of experiments, the extract from *P. eryngii* was heated for 5 min at 90 °C, cooled, and added to the lignin solution in the same experimental conditions described above. Control incubations were carried out either without lignin (Ext-C or Lac-C, respectively) or without enzymes (Lig-C).

### 3.6. Total Phenolics

The total phenolic (TP) content of samples was determined spectrophotometrically with the Folin–Ciocalteu reagent, with some modifications [37]. Briefly, 0.1 mL of the extracts was mixed with 0.5 mL of Folin–Ciocalteu reagent (diluted 10 times with water), and after 2 min, 0.4 mL of 7.5% sodium carbonate was added. After 90 min of incubation at room temperature in the dark, the absorbance was measured at 765 nm. The total phenol content was expressed as μg of gallic acid equivalents (GAEs)/mL.

### 3.7. Reducing Capacity

For the total reducing capacity (TRC) assay, 0.1 mL of the samples was mixed with 0.5 mL of 0.2 M sodium phosphate buffer at pH 6.6 and 0.5 mL of 1% potassium ferricyanide. After 30 min of incubation at 50 °C, 0.25 mL of 20% trichloro acetic acid was added to the mixture and then centrifuged (10 min at 4000× *g*). Afterwards, 0.5 mL of the supernatant was mixed with 0.5 mL of distilled water and 0.1 mL of 0.1% ferric chloride. The absorbance was read at 700 nm [32]. The total reducing capacity was expressed as μg of gallic acid equivalents (GAEs)/mL.

### 3.8. DPPH and Hydroxyl Radical Scavenging Activity

For the DPPH assay, 0.1 mL of samples at different concentrations was added to 0.7 mL of 0.2 mM 2,2-diphenyl-1-picrylhydraziyl (DPPH) in ethanol and incubated at room temperature for 30 min in the dark [32]. The absorbance was measured at 517 nm. For the hydroxyl radical-scavenging assay, the reaction mixture was prepared by adding 0.05 mL of 18 mM salicylic acid, 0.1 mL of sample, 0.1 mL of 9.1 mM heptahydrated ferrous sulfate, and 0.65 mL of water, in that order. Then, 0.6 mL of 8.8 mM H_2_O_2_ was added, and, after incubation at 37 °C for 30 min, the absorbance was measured at 510 nm. The scavenging activities were expressed as IC_50_, where IC_50_ values indicate the sample concentration (μg/mL) required to inhibit (scavenge) 50% of DPPH or OH free radicals. Radical-scavenging activity (%) = [(Abs_control_ − Abs_sample_)/Abs_control_] × 100. Gallic acid was used as the positive control.

### 3.9. MTT Viability Assay

The effect of the different lignin solutions was tested for cell viability on DI-TNC1 cells using the MTT [3-(4,5-dimethylthiazol-2-yl)-2,5-diphenyl tetrazolium bromide] assay as reported by Latronico et al. [38]. The DI-TNC1 cell line (ATCC CRL-2005) was acquired and authenticated from the ATCC (www.lgcstandards-atcc.org, (accessed on 21 November 2024)). Briefly, confluent cells plated in serum-free DMEM in a 96-well plate were treated for 20 h with Ext-mix and Lac-mix, Ext-C, Lac-C, or Lig-C, at concentrations ranging from 10 to 500 µg/mL; then, the culture medium was removed, and cells were incubated for 2 h at 37 °C and 5% CO_2_ with 0.5 mg/mL of MTT. At the end of the incubation, the culture medium was removed and the formazan crystals in the cells were solubilized with absolute ethanol. The amount of the formazan product was determined by optical absorbance at 545 nm with a reference wavelength of 690 nm. Cell viability was expressed as percentage of the negative control (ctrl), represented by untreated cells, which was set at 100%.

### 3.10. Intracellular Reactive Oxygen Species Detection

The detection of reactive oxygen species (ROS) in DI-TNC1 cells was performed as reported by Latronico et al. [39]. Briefly, confluent DI-TNC1 cells, seeded in 96-well plates, were pre-treated for 2 h with Ext-mix, Lac-mix, Ext-C, Lac-C, or Lig-C, at concentrations ranging from 1 to 100 μg/mL, then loaded with 10 μM of the fluorescent probe 2′,7′-dichlorofluorescein diacetate (DCFH-DA) in phenol red–free DMEM. After 30 min of incubation at 37 °C, H_2_O_2_ at a final concentration of 500 μM was added in each well. The negative control (CTRL) was represented by cells treated only with DCFH-DA. The positive control was represented by cells treated only with H_2_O_2_. The fluorescence intensity of cells was measured after 30 min of incubation at 37 °C, through a spectrofluorimetric analysis at 525 nm under excitation at 485 nm in a microplate reader (Cytation 3 Imaging Reader; Bio Tek, Winooski, VT, United States). The results were normalized to cell viability, and ROS production was expressed as a relative percentage of photoluminescence intensity (PLI) versus the positive control (H_2_O_2_) using the following equation: % ROS production = (PLI_sample_/PLI_H2O2_) × 100.

## 4. Conclusions

Lignin represents an enormous potential, largely unexploited, as a source of compounds that can serve as building blocks for products with high added value. A prerequisite for lignin valorization is represented by its depolymerization, which is still an interesting challenge for the industrial research.

Compared to chemical degradation, the biological depolymerization of lignin offers several economic and environmental advantages. Mushrooms represent the most important players in lignin degradation, by producing oxidative enzymes, such as as peroxidases and laccases.

In this study, an aqueous extract obtained from the mushroom *P. eryngii* was used to evaluate the antioxidant properties of the products derived from the enzymatic degradation of lignin. The use of an aqueous extract offers several advantages when compared to the use of a single purified ligninolytic enzyme or mixtures of them. In fact, the aqueous extract is simple to prepare, taking advantage of the synergic action of several enzymatic activities and allowing us to reduce the costs, compared to those incurred for the use of commercial enzymes. The results of this study showed that the enzymatic activities present in *P. eryngii* extract successfully improved the antioxidant performance of lignin through the release of phenol compounds. Furthermore, it was also demonstrated that the compounds derived from the biodegradation of lignin by the mushroom extract do not affect cell viability or inhibit the H_2_O_2_-induced ROS production, thus underscoring their potential for the food industry and for biomedical applications. In conclusion, the use of the *P. eryngii* extract for the bioprocessing of lignin could provide an efficient and ecofriendly approach to obtain lignin derivatives potentially useful for value-added applications in antioxidant research.

## Figures and Tables

**Table 1:** Total phenolics, reducing capacity, and antioxidant activity of samples.

	Total Phenolics (μg GAEs/mL)		Reducing Capacity (μg GAEs/mL)		Radical-Scavenging Activity (IC_50_ μg/mL)			
					DPPH		Hydroxyl	
sample	t_0_	t_18_	t_0_	t_18_	t_0_	t_18_	t_0_	t_18_
Ext-C	62.2 ± 3.7	58.4 ± 2.6	54.4 ± 4.2	47.3 ± 5.1	nd	nd	nd	nd
Lac-C	nd	nd	nd	nd	nd	nd	nd	nd
Lig-C	525.3 ± 7.6	483.6 ± 26.9	326.5 ± 16.2	355.3 ± 34.0	88.2 ± 5.9	92.8 ± 2.1	79.2 ± 3.5	77.7 ± 5.1
Ext-mix	528.2 ± 10.1	* 945.5 ± 15.7	366.1 ± 15.9	* 656.9 ± 22.8	91.2 ± 5.5	* 27.4 ± 3.7	76.3 ± 1.7	* 35.9 ± 4.4
^tt^Ext-mix	513.6 ± 9.7	527.4 ± 20.6	370.2 ± 11.5	366.8 ± 14.4	94.3 ± 8.1	92.22 ± 5.1	78.2 ± 5.5	77.9 ± 3.7
Lac-mix	514.9 ± 7.3	* 594.8 ± 9.5	312.7 ± 17.8	333.0 ± 23.2	87.8 ± 3.4	* 62.5 ± 2.8	81.1 ± 3.3	* 64.3 ± 2.9
Gallic acid (positive control)					5.3 ± 0.3		3.4 ± 0.2	

^tt^Ext-mix: corresponds to lignin incubated with a thermally treated extract from *P. eryngii*. Values correspond to the mean ± SD of two experiments performed in triplicate (n = 6), except for gallic acid (n = 3). Asterisks indicate a statistically significant difference in comparison to the baseline value (t_0_) (one-way ANOVA followed by Tukey’s post hoc test; *p* < 0.05).

**Table 2:** The enzymatic activities detected in the aqueous extract from *P. eryngii* fruiting bodies.

Laccase (U/mL)	Peroxidase (U/mL)
213.0 ± 5.8	52.2 ± 2.2

Values are reported as mean ± SD of two independent experiments performed in triplicate (n = 6).

## Data Availability

Data are contained within the article.
